# Physical-mental health and awareness of HIV/STIs among men who have sex with men in selected cities of India

**DOI:** 10.1186/s12889-023-15202-z

**Published:** 2023-02-13

**Authors:** Suraj Pal, Praveen Kumar Pathak, Margubur Rahaman

**Affiliations:** 1 Department of Geography, Delhi School of Economics, University of Delhi, New Delhi, 110007 India; 2 Department of Geography, Faculty of Natural Sciences, Jamia Millia Islamia (Central University), New Delhi, 110025 India; 3 Department of Migration & Urban Studies, International Institute for Population Sciences (IIPS), Mumbai, 400088 India

**Keywords:** Gay, Bisexual, Homosexuality, MSM, LGBT

## Abstract

**Background:**

Men who have Sex with Men being sexual minorities are a vulnerable section of society and are at greater risk of ill-treatment if they are *‘out of the closet’* regarding their sexuality. Previous evidence suggests that they experienced heightened susceptibility to physical and mental illnesses owing to widespread discrimination and victimization across different walks of life, particularly in developing countries. However, there is a paucity of sound data and scientific understanding related to linkages between physical-mental health and awareness about Human Immunodeficiency Virus/Sexually Transmitted Infections among Men who have Sex with Men in the Indian context.

**Methods:**

Using a mixed-method approach, the present study examined the association between physical-mental well-being and awareness of comprehensive Human Immunodeficiency Virus/Sexually Transmitted Infections/relevant health behaviours based on primary data collected from 300 respondents from six selected metro cities in India. Descriptive statistics, chi-square test and binary logistic regression model have been used for the quantitative data analyses. In-depth interviews were conducted to contextualize and appreciate the substantive meanings and significance coming out from the quantitative results with the lived experiences of the study respondents.

**Results:**

Finding suggests that one-fourth of the respondents were experiencing long-term illnesses while close to four out of ten respondents experienced short-term illnesses. About one-third of the respondents experienced disordered eating behaviour and mood swings. Close to one-fifth of the respondents reportedly contemplated suicidal thoughts. Awareness related to Sexually Transmitted Infections and preventive measures related to Human Immunodeficiency Virus risk was considerably low among Men who have Sex with Men.

**Conclusion:**

Awareness about sexual and reproductive health issues among Men who have Sex with Men needs to be strengthened to curtail the disproportionate risk and vulnerability of Human Immunodeficiency Virus and Sexually Transmitted Infections. The public healthcare system needs to be sensitized and upgraded to cater user-friendly quality healthcare services, without any discrimination against sexual minorities including Men who have Sex with Men. Furthermore, generating public awareness about gender and sexuality-related matters and reducing stigma and discrimination is critical for achieving the health-related sustainable development goals in India without leaving no one behind.

## Background

The United Nations mandated Sustainable Development Goals are closely related to sexual minorities. Goal 3, which deals with “Ensuring healthy lives and promoting well-being for all at all ages” is particularly relevant for the LGBT + community. This goal aims to eradicate epidemics like HIV/AIDS, tuberculosis, malaria, hepatitis and other communicable diseases [1]. Previous studies have indicated that the prevalence of HIV, STIs and other diseases is higher among MSM than among the general population [2–6]. Awareness of the risk of acquiring HIV is limited and the use of condoms is not universal among MSM [7, 8]. Men who are involved in selling sex are at greater risk of getting infected and transmitting HIV to their non-MSM partners, which is blurring the distinction of transmission of HIV between non-heterosexual and heterosexual relationships in India [9, 10]. Morbidity due to preventable diseases (pneumonia, influenza, chronic liver diseases and cirrhosis, hypertensive heart diseases etc.) is also greater among MSM than heterosexual people [11].

Furthermore, MSM living with non-HIV chronic illnesses, face diverse psychological concerns due to multiple stigmas and live a socially invisible life [12]. According to Minority Stress Theory, the LGBT + community including MSM is vulnerable to mental as well as physical illnesses resulting from psychological stress [13]. Ever-going victimization, internalized stigma, unaffordable health services, obesity and a sedentary lifestyle are significantly responsible for poor physical health and mental illnesses among MSM [14, 15]. Health disparity among MSM is dependent upon the availability of health services including knowledge, support and position in society [16–18]. These factors play determining roles in the well-being of subgroups of the MSM community differently.

The depressive symptoms are positively associated with feminine identity among MSM individuals because the nonconforming gender actions and the effeminate nature of these individuals make them vulnerable to stigma in Indian society [5, 19]. The prevalence of suicide is higher among MSM individuals than among their heterosexual counterparts [20, 21]. The utilization of health services is affected by stigma and disclosure of sexuality in public. Whitehead et al. [22] suggested that educating people and service providers and promoting LGBT + inclusion in health services spaces can reduce the stigma related to alternative sexuality.

### Objectives of the study

This study aims to assess the association between physical-mental health and awareness about HIV/STIs behaviour among MSM in selected metro cities of India. The specific aims of this paper are following:

- To examine the physical-mental health status of MSM and understand its association with sexuality.
- To analyse the level of awareness regarding HIV and STIs among MSM and understand the role of socioeconomic and demographic factors affecting HIV testing.

## Methods

### Study area and sample size

This mixed-method cross-sectional study was conducted in six metro cities of India; Delhi, Mumbai, Kolkata, Bengaluru, Ahmedabad and Lucknow. These cities were selected for the convenience of the study as there are a greater number of dating app users. All these cities represent different states of India, located in different geographical spaces and encapsulate the socio-cultural and economic diversities of the country. Considering various factors such as; (a) the non-response rate of respondents to be a part of this study, (b) an extensive questionnaire consisting of questions from diverse dimensions of MSM, (c) restricted time allotted for data collection and (d) availability of limited financial resource as part of doctoral dissertation etc., it seemed rationale that 300 respondents would be reasonable to conduct an exploratory study using a mixed-method approach on such a sensitive topic. So, it was decided that 50 respondents will be selected from each metro city.

### Sampling design and selection of the respondents

Non-probability community venue sampling [23] technique was employed to recruit study respondents. This was a feasible sampling mechanism to target self-identified MSM respondents who were using the dating app. This sampling design was convenient and purposive, which makes the present study exploratory in nature. The inclusion criteria of respondents were; (a) respondents must be 18 years old or above and (b) they identify themselves as an MSM individual. Respondents were recruited through a popular dating app ‘Grindr’, which is a location (GPS) based social networking and online dating application for gay, bisexual, transgender and queer people. This app was used as a venue to find probable respondents. First of all, an account was created after downloading the app from the Google play store. The motive for joining the platform was stated clearly in the bio/description section that ‘this account is created solely for research purposes on issues related to MSM’. Initial screening for the selection of respondents was done on the basis of description, sexual orientation, sexual position etc., which was given on the user’s account. Respondents using the dating app nearby railway stations, bus stands, universities, markets and other such places were communicated through text and audio messages. The response rate of dating app users for participating in the survey was around 30 per cent when they were contacted virtually. After the virtual connectivity, they were requested to meet physically and participate in the study. The response rate increased to around 99 per cent when they met face-to-face for a personal interview. All the respondents categorized themselves as gay or bisexual depending upon their engagement in sex with only men, or with men and women both respectively. Considering this distinct classification of the sexual orientation of MSM, 25 gays and 25 bisexuals were selected from each city.

### Data collection and analysis

A pilot study was done in Delhi to check the robustness of the data collection instrument. After this, a semi-structured schedule was used to collect data between June 2019 to February 2020. The questionnaire included many objective and subjective questions to cover different dimensions of the lives of MSM. All questions were asked in Hindi or English. Only quantitative data were collected from 240 respondents while both qualitative and quantitative data were collected from 60 respondents (10 from each city and in the same session), which took 35 and 85 min on average respectively to complete in-depth interviews. Extreme incidents/experiences (like sexual encounters with 100 + people in the last 12 months, failed suicide attempts, abducted or raped by other MSM individuals etc.) were factors for considering qualitative data collection through in-depth discussion. Respondents were not given any monetary compensation for participating in this study. All the illnesses that were considered for this study were self-reported by the respondents. Short-term illnesses were referred to as any temporary sickness the respondents have had in the past week of in-depth interviews, which were defined as illnesses, which go away quickly or make the person sick for a relatively shorter period of time; for example common flu, fever, headaches etc. Long-term illnesses were defined as illnesses, which remain for longer periods of time; for example HIV/AIDS, diabetes, pulmonary diseases etc. Respondents were explained in detail about the research objectives, different sections of the survey instruments and any possible risk related to their participation in the study. Verbal informed consent from the study respondents was sought and only those who consented were recruited for the study as per the approval from the ethics committee. Privacy and confidentially have been ensured for all the study respondents. The study participants were apprised that the information sought from them shall be used purely for the research and publication. Respondents who were engaged in risky sexual behaviours were requested to get tested for HIV and STIs and they were given references of government hospitals and some NGOs. Respondents, who were experiencing mental distress and were not taking any professional help, were suggested to seek professional medical services at the earliest. Qualitative analysis was done based on information collected through in-depth interviews to substantiate the quantitative data and provide meanings and substantive context by identifying specific incidents, important phrases and particular words (translated into English). Univariate and bivariate techniques, chi-square test and binary logistic regression were used for quantitative analysis. HIV testing was used as a dependent variable while age, sexual orientation, sexual position, disclosure of sexuality, marital status, education level, religion, job collars, annual family income and residential city were used as independent variables for predicting the likelihood of HIV test among MSM. The result of the binary logistic regression model is presented in the form of odds ratios with 95 per cent confidence interval. This analysis was carried out using SPSS software (V20).

### Definition of predictor variables and category for binary logistic regression

#### Sexual orientation

It was referred to as romantic attraction towards other people. Sexual orientation was categorized into gay (one who is romantically inclined towards the person of the same sex only) and bisexual (one who is romantically inclined towards the person of both sexes i.e. male and female) in this study [24].

#### Sexual position

It was referred to as a position opted by an individual during lovemaking. The sexual position is categorized into top (one who is the dominant male or involved in the active role during sex), bottom (one who is the submissive male or involved in the passive role during sex), versatile (one who can play both roles i.e. top and bottom) and side (one who is not into penetrative sex during same sex sexual activity) in this study [25].

#### Job Collar

It was referred to as the primary profession of the respondents. Job collar was categorized into white (includes respondents who were salaried professionals), blue (includes respondents who were engaged in manual labour or paid on an hourly basis for the work), pink (includes respondents who were employed in care-oriented sectors) and others (includes respondents who were student, not working/unemployed) in this study.

#### Annual Family Income

It was referred to as the annual family income of respondents in the Indian Rupee. Annual family income was categorized into low (400,000 and below), middle (400,001–1,000,000) and high (1,000,001 and above) income groups in this study.

## Results

The basic demographic and socioeconomic profile of the respondents of this study is provided in Table 1.

**Table 1:** Socio-economic and demographic profile of study respondents

Background Characteristics	Percent	Sample (n)
Age (in completed years)		
18–23	29.0	87
24–30	40.3	121
31 and above	30.7	92
**Sexual Orientation**		
Gay	50.0	150
Bisexual	50.0	150
**Sexual Position**		
Top	34.0	102
Bottom	25.3	76
Versatile	34.7	104
Side	6.0	18
**Out about Sexuality**		
Yes	27.7	83
No	72.3	217
**Marital Status**		
Never Married	76.3	229
Currently Married/Divorced/Widowed	23.7	71
**Education level**		
Primary and below	5.3	16
Secondary and Higher Secondary	27.7	83
Graduation and above	67.0	201
**Religion**		
Hindu	71.3	214
Muslim	18.0	54
Christian	4.3	13
Others	6.3	19
**Job collars**		
White	33.0	99
Blue	19.3	58
Pink	10.0	30
Others	37.7	113
**Annual Family Income**		
Low Income	35.0	105
Middle Income	37.3	112
High Income	27.7	83
**Residential City**		
Ahmedabad	16.7	50
Bengaluru	16.7	50
Delhi	16.7	50
Kolkata	16.7	50
Lucknow	16.7	50
Mumbai	16.7	50
**Total**	**100.0**	**300**

### Physical health

Data in Table 2 show that the frequency of experiencing short-term illnesses and long-term illnesses was highest among bottom (44.7 per cent) and versatile (31.7 per cent) respondents respectively. One-third of the total respondents were experiencing disordered eating behaviour (a condition of either eating more or less than required). P-value shows that experiencing disordered eating behaviour was significantly associated with sexual position. The frequency of going gym and involvement in sports activity was lowest among bottom and these physical activities were significantly associated with sexual position.

**Table 2:** Descriptive statistics of physical health characteristics by sexual position of respondents (N = 300)

Physical health characteristics	Sexual Position (in %)				Total (in %)	*p-value*
	Top	Bottom	Versatile	Side		
Experiencing any short-term illness	33.3	44.7	42.3	33.3	39.3	*0.373*
Experiencing any long-term illness	19.6	30.3	31.7	11.1	26.0	*0.081*
Experiencing disordered eating behaviour	20.6	42.1	37.5	44.4	33.3	*0.000*
Go to Gym at present	39.2	3.9	28.8	11.1	25.0	*0.008*
Involved in any sport activity	30.4	9.2	25.0	27.8	23.0	*0.008*

*Chi-square tests were used to obtain P-value*

#### Correlation of short and long-term illnesses among MSM

A significant number (79.5 per cent; p-value ≤ 0.000) of respondents interviewed for this study facing long-term illnesses, were also facing short-term illnesses. Such a case of one of the respondents (a 54-year-old software developer from Delhi), is quoted below:

*I am a patient with diabetes, there’re so many restrictions on my diet already. I can’t eat what my taste buds like! I know it has nothing to do with my sexuality but you need to be happy to live long. Frankly speaking, I don’t have any materialistic or non-materialistic happiness in my life. Life has become a burden now. Thank God that I’m a closeted gay; otherwise, I would have committed suicide long ago. I live alone in Delhi. Now living this monotonous life all alone, there’s no one to take care of me even when I’m sick. I think I’m getting mad, “life me ab kuchh bachha nahi yar (there’s nothing left in my life now)”. I’m tired of this life, don’t want to live more!*

#### ‘Look good syndrome’ among MSM

Majority of the respondents admitted that “looking good in the community is like getting a ticket to acceptance by almost everyone”. This ‘look good syndrome’ is characterized by typical body objectification, which includes fair colour, toned shape of the body, muscle power and visibly looking masculine features. “Masculinity is worshipped here and that is why most of the MSM individuals tend to become a *Top* if they are capable to perform as an active partner or able to do insertive anal intercourse”, said a 24-year-old post-graduating student from Kolkata. He added further, “Not so fair colour of the body can be compensated with a good gym-built personality”. A 21-year-old respondent from Delhi, who is a college-going student revealed, why he is so much passionate about building muscles. The following narrative shows his view on this:

*Going to the gym has become an integrated part of my life, not because I don’t find myself attractive without six-pack abs but ‘they’ (their customers) like it on me! My family is not able to bear expenses for my luxurious lifestyle, I like going to clubs and disco, wear branded attires, organize party and what not! But I need money for this. Many people found me cute initially and offered me money for physical intimacy, but I knew I’m not making that much money compared to other gym-toned boys. Finally, I joined the gym so that I can sell my look to my clients and I’m happy that it is working fine. Now I feel I’m independent at this early age. “Paisa kamana zaruri hai, kaise kamate ho isse fark nai padta! (Earning is important, doesn’t matter how you make it!)”.*

#### Low level of engagement in outdoor sports among MSM

Approximately 23 per cent of respondents told that they were actively involved in any of the outdoor sports. However, most of the respondents agreed that it is the activity, one should engage in to be physically fit. They knew the benefits of sports in building social connections too but some could not do it because of the modern sedentary lifestyle. Some respondents did not get enough time for this. A few admitted that they wish to do so but they cannot face verbal or sometimes even physical bullying by others around. A 19-year-old student from Kolkata shared his experience regarding this:

*I cannot take those nasty jokes on me! First of all, they (other boys) don’t make me even feel that I am a boy and I can play with them. And when they include me in the team on occasion when there is a shortage of team players, they call me with derogatory words to pass the ball, mimic my feminine voice, make some absurd comments and laugh at me. In that stigmatized scenario, if you expect someone to play well then it is beyond imagination. “Are tum khelne ka baat to chhodo, waha khada rahna bhi mushkil ho jata hai (forget about playing, it becomes challenging even to stand there)”. When this drama kept on increasing and getting unbearable, I stopped participating in outdoor sports activities.*

#### Abnormal eating behaviour among MSM

One-third of the total respondents reported experiencing disordered eating behaviour. This impacts the body mass ratio, resulting in making them obese (for people who eat more) or skinny (for people who eat less). This condition was the result of more psychological than physical aspects as reported by respondents. Too much thinking and stressful life were some of the factors responsible for disordered eating behaviour, which most of the respondents recognized. A 28-year-old photographer from Bengaluru, shared his experience, which is stated below:

*There’s always a sense of lowliness when I judge my physical personality. It’s not masculine enough to be called a man in Pubic! This feeling makes me feel shattered, I feel to be very weak from inside and outside. It directly impacts my food habits… I can’t eat more. I feel my stomach is full after eating the amount of food that a 10-year-old kid consumes. I got all the tests done which doctors prescribed me and then they said I’m taking too much stress. I should avoid over-thinking and be optimistic. Hahaha (laughing)… to be honest, I’ve nothing to be happy in this life!*

### Mental health issues faced by MSM

As discussed above, many physical health outcomes are dependent upon the mental health status of an individual. This segment of the study attempts to understand the frequent mental illnesses among MSM based on their lived experiences. Data in Table 3 show that the frequency of feeling isolated or disconnected and getting anxious or uneasy or nervous was higher among gay than bisexual respondents. More than one-third of total respondents got negative or pessimistic thoughts and this mental characteristic was significantly associated with sexual orientation. Around 22 per cent of gay respondents thought of committing suicide while 17.3 per cent of total respondents thought that they were inferior to others (heterosexual people). Bisexual respondents felt more pressure to behave like straight people. P-value shows a significant association between feeling pressure to behave like straight people and sexual orientation. Data in Table 4 show that six out of seven mental health parameters of respondents who participated in this study are significantly associated with long-term illnesses they are experiencing.

**Table 3:** Descriptive statistics of mental health characteristics by sexual orientation of respondents (N = 300)

Mental health characteristics	Sexual Orientation (in %)		Total (in %)	*p-value*
	Gay	Bisexual		
Feel isolated/disconnected	31.3	12.7	22.0	*0.000*
Feel anxious/uneasy/nervous	28.0	18.7	23.3	*0.001*
Get negative/pessimistic thoughts	40.0	30.7	35.3	*0.000*
Experiencing mood swings	36.7	34.0	35.3	*0.815*
Suicidal ideation	22.0	16.0	19.0	*0.000*
Consider yourself inferior than others	22.0	12.7	17.3	*0.015*
Under pressure to behave like straight people	28.7	38.7	33.7	*0.003*

*Chi-square tests were used to obtain P-value*

**Table 4:** Descriptive statistics of mental health characteristics of respondents in correlation with long-term illness (N = 78)

Mental health characteristics	Experiencing long-term illness (in %)	*p-value*
Feel isolated/disconnected	48.7	*0.000*
Feel anxious/uneasy/nervous	46.2	*0.000*
Get negative/pessimistic thoughts	66.7	*0.000*
Experiencing mood swings	48.7	*0.007*
Suicidal ideation	38.5	*0.000*
Consider yourself inferior than others	30.8	*0.000*
Under pressure to behave like straight people	43.6	*0.098*

*Chi-square tests were used to obtain P-value*

#### Isolation or loneliness among MSM

Approximately one-fourth of total respondents said that they often feel disconnected from the straight (heterosexual) community and their social life interaction with them is as low as possible. They cope with the emotional trauma on their own. This isolation makes them vulnerable to other psychological problems. A 20-year-old student from Lucknow discussed this issue, which is narrated following:

*Since I got conscious about what I want (the moment I realised I am gay), I tried to isolate myself from my family. Though I never came out to them about my sexuality, I’m very sure they will never understand this concept. Their orthodox mentality is way beyond understanding these liberal issues. The same thing I’ve been doing in my school too. I fear if anyone come to know about it, they’ll discard me from their friend zone, so it’s better to be prepared and not indulge with homophobic people (probable) in the first place!*

#### Anxiety or nervousness among MSM

Respondents informed that they are under pressure to behave like straight people in public places. This act of pretending all the time makes them very uneasy. Many a time they behave very abnormally and the situation gets awkward. This ongoing real-life acting makes these sexual minorities (especially gays) anxious and nervous. The degree of anxiety gets stronger with each incident handled unexpectedly. However, the anxiety level was low among respondents who were resilient and had a coping mechanism. A 24-year-old tailor from Lucknow, shared his experience, the narrative is following:

*In our country it’s a sin to be gay but worse than that is when people come to know about you. So I try my best to not give any hint to people that I’m a homosexual but this super consciousness creates unwanted panic in my head. Once I was in the college canteen, I felt that a group of boys are staring at me. I got so nervous that I started walking very fast to avoid any joke upon me, I could not maintain balance and I fell. What a sheer embarrassing moment it was! And it’s not the only incident, there are many like this. The more I become conscious about people’s perception, the more I sink into anxiety.*

#### Negative or pessimist thinking among MSM

Few MSM individuals themselves thought that homosexuality is an inferior trait to heterosexuality. In a homophobic society, they often witnessed only demoralizing comments regarding sexual minorities. They have been treated like lower-class citizens. Films and social media platforms have contributed toward pejorative portrayals of homosexual communities. The negative environment led to internal homophobia among MSM. A 22-year-old dance teacher from Ahmedabad shared his part of the story in the following narrative:

*I live with my maternal uncle since my mother father passed away. Everything was going good until (3 years ago) some well-wishers (elder boys in my locality) of my uncle informed him that I’m socializing with folks, who are identified as gays. I was given a strict warning by my uncle and aunt that if they come to know one more incident like this, they will kick me out of their home. This led me to choose the shelter of my custodian uncle because I had no place to go and I was not fully economically independent. I’m so much scared of that incident that I don’t like the gay part of mine. I’ve started hating myself for this. I wish sexuality could be a choice…*.

#### Mood swings among MSM

More than one-third of the total respondents admitted that they often experiencing mood swings (sudden changes in mood). Depending upon the incidents throughout their life, the mood swing ranges from depressive lows to manic highs. This was more frequent among respondents having hypersexual desires. A 32-year-old chartered accountant from Mumbai shared his experience:

*I feel very helpless at times of stress. Daily struggling with acceptance or denial of my sexuality makes me move to and fro to the extremes of it. One moment I decide that I will give up this ‘homo life’ forever, refrain myself from meeting gay friends, stop using Grindr but the next moment I do just the opposite of it, I meet people through Grindr and indulge in random sex. I am so helpless! Though I don’t consider it to be a mental illness, I know many friends who are going through the same phase of denial about their sexuality (homosexuality). All of us don’t want to accept that we’re ‘Homos’, still we indulge in this swamp.*

#### Suicidal thoughts among MSM

About 19 per cent of respondents reported that they often have/had suicidal thoughts in their minds with the particular reason of being a sexual deviant. Gay-identified respondents were more likely to report suicidal thoughts as compared to bisexual respondents, in this study. The cope up mechanism was found to be low among gays, which makes them think of ending their lives. Verbal and physical victimization against the MSM community and within the MSM community made male sexual minorities vulnerable to think about suicide. Many respondents shared their experiences related to suicide attempts, two of which are narrated below:

*I came out to my parents about my sexuality at age of 22. They were shocked. It was a mistake I guess, it was too early to tell them all this. They thought it is a disease, they took me to the doctor, I told them medication will do nothing as it’s not an illness. You won’t believe… they took me to a Baba (saint), who gave me Bhabhut (ash), which I needed to consume to get rid of homosexuality. There was always pressure from my parents to act like a man, I had to listen to lectures on how to walk, how to talk, how to laugh and many other things! One day I decided to end my life by cutting the vein of one of my wrists. (a 29-year-old fashion designer from Mumbai)*

*I realised it very late (at age of 26) that I’m into men! I was in a relationship with two girls earlier but it didn’t work. Then he came into my life… for the first time, I felt complete. I was so happy with him… he shifted with me to my rented flat. Everything seemed to be so nice for six-seven months and then one day he told me he’ll have to move to another location. I couldn’t understand the reason behind it. I told him I have no issue moving with you anywhere! He said: “You can’t come with me”, I asked: “why so?” for which he replied: “I was never serious about for relationship but I guess you’re going too deep into this. Consider all that a time pass and forget me!” and he left… After spending so much of my time, dedication and money on the relationship, it took him a moment to break up with me! I couldn’t bear the pain of this broken relationship, so tried to kill myself by gulping plenty of sleeping pills. (a 30-year-old architect from Ahmedabad)*

#### Inferiority complex among MSM

Many respondents used to compare their lives with people of the straight community. This brought unnecessary inferior feelings among a few individuals. Many respondents believed that they could have done better in their professional life if they were straight. Productivity is compromised in the professional field due to exerted or perceived discrimination related to the homophobic culture present around sexual minorities. Few respondents believed that their sexuality is a hindrance in their careers. A 25-year-old data operator from Mumbai shared his experience, which is quoted below:

*I always feel very insecure about my personality when I see straight guys. Deep within, I know that I’m inferior and they are superior. From school life to office life, I’ve always missed that confidence that makes an individual perform his best. Most of the time I spend my day and night struggling with odd perceptions related to probable consequences of being a homosexual! Comments/jokes related to gays, made at school or office hit my inner person directly. I feel as if there is a thief inside me… but coming out will make the situation worse I guess. Battle with my profession is secondary in the list of problems of my life, first is the perennial struggle to be alive as a gay on daily basis.*

### Diseases and utilization of health services among MSM

Respondents who participated in this study were asked about some frequent diseases among MSM. This information was useful in understanding the comprehensive and correct knowledge of diseases among the community members, which reveals HIV and STIs testing attitudes, the use of some preventive measures and the utilization of health services. Data in Table 5 show that only half of the total respondents had ever been tested for HIV. About 43 per cent of respondents had never heard of Syphilis/Gonorrhoea/Herpes. Awareness regarding oral HPV was very low among respondents while the majority (62.7per cent) of the respondents had never heard of PEP and PrEP and only 3.7 per cent of respondents were using it as a preventive measure against HIV.

**Table 5:** Percent distribution of awareness and testing for selected diseases and use of preventive measures related to HIV among respondents (N = 300)

Diseases and Preventive measures	Never heard	Heard only	Tested
HIV/AIDS	1.3	48.7	50.0
Syphilis/Gonorrhoea/Herpes	43.0	32.3	24.7
Oral HPV	66.0	25.3	8.7
PrEP/PEP	62.7	33.7	3.7

Data in Table 6 show that 8.4 per cent of respondents thought that HIV can be transmitted by eating with an HIV-positive person while 12.8 per cent of respondents said that a healthy-looking person cannot be infected with HIV. About 7.1 per cent of respondents said that correct and consistent use of condoms cannot prevent HIV while 13.9 per cent of the respondents had no idea about it. More than half (54.7 per cent) of the total respondents had either no awareness or had the incorrect information about ART. This incorrect information regarding ART included: (a) “No treatment can save you/increase longevity once you are HIV positive”, (b) “You have to take many pills every day, which is not feasible” and (c) “The medicines do more harm than good to your body”. About 71.3 per cent of respondents did not know about the window period of HIV.

**Table 6:** Percent distribution of comprehensive knowledge regarding HIV among respondents (N = 300)

Question related to HIV	Yes	No	Don’t Know
Can HIV be transmitted through unprotected anal intercourse?	94.3	2.0	3.7
Can HIV be transmitted by eating with an HIV positive person?	8.4	80.4	11.1
Can a healthy-looking person be infected with HIV?	65.5	12.8	21.6
Can HIV be prevented by consistent and correct condom use?	79.1	7.1	13.9
Can ART help HIV positive person to live longer?	45.3	12.8	41.9
Do you know about window period of HIV?	28.7	71.3	---

#### Awareness and testing of HIV among MSM

A significant number of respondents have at least heard of HIV and only half of the total respondents had ever been tested for it. The majority of the respondents knew that (a) HIV can be transmitted through unprotected anal intercourse (b) the virus cannot be transmitted by eating with HIV positive person (c) a healthy-looking person can be infected with HIV and (d) HIV can be prevented by consistent and correct condom use but many respondents had no fair knowledge of ART. A 42-year-old shop owner from Ahmedabad shared his perception regarding HIV test, which is narrated below:

*I don’t think I’m at risk of HIV! I always use a condom during sex with boys. I meet people who look healthy with good physique. Rest if I start testing for HIV, I’ll have to do it every now and then… My sexual urge never ends and so my hunt for boys (for random sex). That’s why I don’t bother much about it. Everyone’s destiny is written before they’re born; if god wants me to die because of HIV, let it be. No one can change it…*.

Data in Table 7 show that the frequency of HIV testing was increasing with the increasing age of respondents and it was significantly associated with age groups. About 55.6 per cent of respondents whose sexual position was side while 39.5 per cent of respondents whose sexual position was bottom, were ever tested for HIV. The rate of HIV testing was more among currently married/divorced/widowed (63.4 per cent) respondents than never married (45.9 per cent) respondents and the p-value shows that there was a significant association between HIV testing and marital status. Frequency of HIV testing was highest among respondents having education qualification graduation and above (59.7 per cent) and HIV testing was significantly associated with education level. HIV testing rate was highest among Christian (61.5 per cent) while lowest among Muslim (46.3 per cent) respondents. About 67.7 per cent of respondents employed in white-collar jobs while 20 per cent of respondents employed in pink-collar jobs had ever been tested for HIV. P-value shows that HIV testing and job collars were significantly associated. There was a consistent increase in HIV testing with the increase in the annual income of respondents and it was 57.8 per cent among respondents belonging to high-income groups. The frequency of HIV testing was highest in Bengaluru (64 per cent) while lowest in Lucknow (32 per cent).

**Table 7:** Socio-demographic differentials in the frequency of HIV testing among respondents

Background Characteristics	Percent	Sample (n)
Age (in completed years)		
18–23	31.0	27
24–30	55.4	67
31 and above	60.9	56
*P-value*	*0.000*	
**Sexual Orientation**		
Gay	45.3	68
Bisexual	54.7	82
*P-value*	*0.106*	
**Sexual Position**		
Top	54.9	56
Bottom	39.5	30
Versatile	51.9	54
Side	55.6	10
*P-value*	*0.193*	
**Out about Sexuality**		
Yes	49.4	41
No	50.2	109
*P-value*	*0.897*	
**Marital Status**		
Never Married	45.9	105
Currently Married/Divorced/Widowed	63.4	45
*P-value*	*0.010*	
**Education level**		
Primary and below	43.8	7
Secondary and Higher Secondary	27.7	23
Graduation and above	59.7	120
*P-value*	*0.000*	
**Religion**		
Hindu	50.0	107
Muslim	46.3	25
Christian	61.5	8
Others	52.6	10
*P-value*	*0.791*	
**Job collars**		
White	67.7	67
Blue	46.6	27
Pink	20.0	6
Others	44.2	50
*P-value*	*0.000*	
**Annual Family Income**		
Low Income	41.9	44
Middle Income	51.8	58
High Income	57.8	48
*P-value*	*0.085*	
**Residential City**		
Ahmedabad	44.0	22
Bengaluru	64.0	32
Delhi	58.0	29
Kolkata	46.0	23
Lucknow	32.0	16
Mumbai	56.0	28
*P-value*	*0.020*	
**Total**	**50**	**150**

*Chi-square tests were used to obtain P-value*

Data in Table 8 show that respondents of the age group 24–30 were 97 per cent (OR: 1.97; CI: 0.96–4.04) more likely to get tested for HIV than respondents of the age group 18–23. The odds of getting tested for HIV were 29 per cent (OR: 0.71; CI: 0.28–1.81) less among respondents whose sexual position was bottom compared to respondents whose sexual position was top. Respondents who were not out regarding their sexuality to family and surrounding, were 56 per cent (OR: 0.44; CI: 0.20–0.96) significantly less likely to get tested for HIV than respondents who were out. The odds of getting tested for HIV were approximately two times (OR: 2.22; CI: 0.91–5.41) more among respondents who were currently married/divorced/widowed compared to respondents who were never married. The odds of getting tested for HIV were 12 per cent (OR: 1.12; CI: 0.53–2.33) more among Muslim respondents compared to respondents who were Hindu. Respondents employed in pink-collar jobs were 84 per cent (OR: 0.16; CI: 0.04–0.58) significantly less likely to get tested for HIV than respondents employed in white-collar jobs. The odds of getting tested for HIV were approximately two times (OR: 2.66; CI: 1.08–6.56) significantly more among respondents residing in Bengaluru than respondents residing in Ahmedabad.

**Table 8:** Adjusted odds ratio predicting likelihood of HIV testing by selected socio-demographic characteristics among MSM

Background Characteristics	Adjusted Odds	95% CI
Age (in completed years)		
18–23®	1.00	
24–30	1.97	0.96–4.04
31 and above	1.76	0.63–4.89
**Sexual Orientation**		
Gay®	1.00	
Bisexual	1.07	0.53–2.16
**Sexual Position**		
Top®	1.00	
Bottom	0.71	0.28–1.81
Versatile	0.93	0.46–1.85
Side	0.77	0.56–2.33
**Out about Sexuality**		
Yes®	1.00	
No	0.44*	0.20–0.96
**Marital Status**		
Never Married®	1.00	
Currently Married/Divorced/Widowed	2.22	0.91–5.41
**Education level**		
Primary and below®	1.00	
Secondary and Higher Secondary	0.51	0.14–1.82
Graduation and above	1.02	0.26–3.97
**Religion**		
Hindu®	1.00	
Muslim	1.12	0.53–2.33
Christian	0.77	0.21–2.80
Others	0.84	0.29–2.41
**Job collars**		
White®	1.00	
Blue	0.45	0.19–1.06
Pink	0.16*	0.04–0.58
Others	0.66	0.31–1.38
**Annual Family Income**		
Low Income®	1.00	
Middle Income	1.01	0.52–1.96
High Income	0.76	0.35–1.66
**Residential City**		
Ahmedabad®	1.00	
Bengaluru	2.66*	1.08–6.56
Delhi	2.06	0.82–5.17
Kolkata	1.28	0.51–3.21
Lucknow	0.62	0.25–1.53
Mumbai	2.16	0.87–5.32

Note. ®= Reference category; *p < .05

#### Awareness and testing of STIs among MSM

Respondents of this study had a low level of awareness regarding STIs. The experience of an 18-year-old worker at a cycle repair shop in Mumbai is narrated below:

*For the last two years, I’m meeting guys using Grindr. I never heard of these words or something (STIs) like that you just stated. That dating app is a platform to meet guys for hook ups only. The conversation starts with your sexual preferences along with face pictures and story ends after you’re done with sex! But yes, I have heard of HIV/AIDS on the dating app itself (in the information section of Grindr and few guys asking about it). One or two men I had sex with, told me: “it doesn’t spread through men to men sex, it happens when you visit brothel houses and do it with women”.*

#### Utilization of health services among MSM

More than half of the total respondents of this study reported that (a) they did not receive satisfactory medical care, (b) there was a delay in getting health services and (c) the cost of the medical services was non-affordable for them. Stigma, discrimination and fear to face the medical staffs including doctors were some of the factors that were reported as obstacles in the early diagnosis of diseases. A 22-year-old UPSC aspirant from Lucknow faced a similar experience that is quoted below:

*HIV test is a must for an individual who’s sexually active with multiple partners! I know the importance of early diagnosis but it is so embarrassing to face the staffs and get the test done. Once I visited a charity hospital in the city, the moment I started enquiring about the fee and procedure of the HIV test at the reception, that gentleman yelled at me and showed me the counter I needed to go. Meanwhile, he was saying “Pata nahi kaise-kaise log aa jate hain, wo bhi itni kam umar me… chhi! (Don’t know what kind of people visit here, that too in such an early age… disgusting!).”*

## Discussion and conclusion

The present study makes a novel attempt using a mixed-method approach to examine the inter-relationship between physical and mental health outcomes and its linkages with the comprehensive knowledge of HIV/AIDS among MSM across selected metro cities in India. Data from the present study confirmed that MSM are vulnerable to various physical and mental health problems [26], which are guided by various factors like psychology, society, behaviour etc. [27]. Significant numbers of respondents in this study experiencing long-term or chronic illnesses were also experiencing frequent short-term illnesses. The findings of another study confirm the association of co-morbidities with chronic illnesses [28]. Researchers [29, 30] have also found a correlation between psychiatric morbidity and chronic diseases, which is one of the findings of this study. Disordered eating behaviour was also frequent among respondents of this study which is similar to findings of other studies [31, 32], where researchers found that disordered eating behaviour is related to a high extent of femininity scores. The findings of Fredriksen-Goldsen et al. [15] validate the findings of the present study that the mental health of MSM is determined by their socio-economic status, lifetime victimization and internalized stigma. Qualitative data of the present study indicated that respondents who had the support of family members and had access to medical help, were able to sail through depressive symptoms. Researchers [5, 33, 34] also found that there is variation in experiences of victimization among subgroups of the LGBT + community, this distinction was evidently reflected in the in-depth interviews of the present study that respondents who identified themselves as feminine, were more victimized than respondents who identified themselves as masculine. Self-doubt, negative attitude towards life, social and interpersonal stressors, fear of getting identified as MSM and rejection by society are some of the reasons which compel individuals to take drastic steps like suicide. Some researchers [35–37] found similar reasons for committing suicide by sexual minorities in their studies.

Physical isolation was adopted as one of the coping mechanisms by MSM while dealing with mental pressure. Johnson and Amella [38] had similar finding that gays and bisexuals withdraw their social connection from family and friends because of not being able to relate with the group. Loneliness, lethargic lifestyle and lack of fitness motivation might harm physical health in the case of many study respondents, this finding is similar to the findings of various studies [38–40]. Discriminatory attributes against MSM and low self-esteem were a few factors for anxiety and pessimistic attitudes among some respondents. This finding is in the same direction as earlier studies, where researchers studied that a complex web of negative views and stigmatization from society make MSM prone to mental illnesses [40–42]. More than one-third of the total respondents reported that they often experience mood swings. Shoptaw et al. [43] found that mood swing among MSM is associated with substance abuse, co-morbidities and risky sexual behaviours. Some health issues like disordered eating behaviour, HIV etc. were frequent among MSM in the present study, studies of other researchers [6, 11, 26, 31, 32] support this finding.

Testing for HIV was 50 per cent among study respondents. The frequency of testing for HIV was similar in other studies [7, 44] conducted in India, where researchers found that HIV testing has an association with the availability of free and comfortable testing locations. Testing and awareness of STIs like Syphilis/Gonorrhoea/Herpes were further low among respondents. The high frequency of STIs is a challenge in lowering HIV incidence, which is a threat to the MSM community [45]. Utilization of health services was reported to be affected by factors like the type of disease, degree of stigma and discrimination by health workers, availability of health services in the local area etc. Alencar Albuquerque et al. [46] studied that the heteronormative attitude of healthcare professionals makes it difficult to deal with the health problems of MSM. The rate of use of preventive measures for HIV i.e. PrEP and PEP was limited to individuals who could afford it, as it is expensive for the majority of the Indian population.

Finally, we report four important take-home messages that family, civil society, formal and informal institutions, government and other stakeholders should ensure a stigma and discrimination-free society for sexual minorities because stigma and discrimination have negative impacts on the overall well-being of sexual minorities. Counselling platforms should be made, which are easily accessible for sexual minorities who need help at times of emotional crisis related to their sexuality to decrease the frequency of suicidal incidents. Rigorous awareness campaigns should be run to encourage sexual minorities (who engage in sexual activities with multiple partners) for periodical testing for HIV and STIs. The public health care system must be trained to cater friendly and quality health services to all sexual minorities without any discrimination.

## Limitations of the study

1. The present study is exploratory in nature and based on a relatively small sample size. The findings from the study are useful to understand the health vulnerabilities across selected urban centres in India. However, given the purposive sampling design using the dating app *Grindr*, the results need to be interpreted with caution and may not be generalizable to all MSM population.
2. The sampling for the quantitative component was purposive and the findings might be limited only to the subset of the population who agreed to be surveyed.
3. For the qualitative phase, those individuals were selected who reported high risks and/or aggravated experiences of trauma (sexual assault, kidnapping) in the quantitative phase as opposed to a “representative” subset of those who responded in the quantitative phase.
4. Findings from qualitative studies should not be viewed from the perspective of generalizability because there are potential biases in the data presented as individuals selected for in-depth interviews have experiences of extremely adverse events than other studies on Indian MSM.
5. The frequency of illnesses was self-reported by the respondents for which neither standardized scales were used for measuring mental health components of the survey nor those illnesses were diagnosed by any professional/medical expert for most of the respondents.
6. The six cities investigated in this paper are very different in their approach to LGBTQ + issues, access to health services, and socioeconomic status indicators. The city-wide differences have not been examined in detail for the study respondents.

## Data Availability

The datasets generated and/or analysed during the current study are not publicly available because of the anonymous nature of it and the privacy concerns of respondents but are available from the corresponding author upon reasonable request.

## Abbreviations

AIDS: Acquired Immune Deficiency Syndrome
ART: Anti-Retroviral Therapy
CI: Confidence Interval
GPS: Global Positioning System
HIV: Human Immunodeficiency Virus
HPV: Human Papillomavirus
LGBT+: Lesbian, Gay, Bisexual, Transgender etc.
MSM: Men who have Sex with Men
PEP: Post-Exposure Prophylaxis
PrEP: Pre-Exposure Prophylaxis
STI: Sexually Transmitted Infection
UPSC: Union Public Service Commission
